# Therapeutic potential of MSCs and MSC-derived extracellular vesicles in immune thrombocytopenia

**DOI:** 10.1186/s13287-023-03323-6

**Published:** 2023-04-12

**Authors:** Feifeng Wu, Zhou She, Cuifang Li, Jueyi Mao, Senlin Luo, Xiaoyu Chen, Jidong Tian, Chuan Wen

**Affiliations:** Department of Pediatrics, The Second Xiangya Hospital, Central South University, Changsha, 410011 China

**Keywords:** Immune thrombocytopenia, Mesenchymal stem cells, Cellular therapy, Extracellular vesicles

## Abstract

Immune thrombocytopenia (ITP) is an acquired autoimmune disease involving a variety of immune cells and factors. Despite being a benign disease, it is still considered incurable due to its complex pathogenesis. Mesenchymal stem cells (MSCs), with low immunogenicity, pluripotent differentiation, and immunomodulatory ability, are widely used in a variety of autoimmune diseases. In recent years, impaired bone marrow mesenchymal stem cells (BMMSCs) were found to play an important role in the pathogenesis of ITP; and the therapeutic role of MSCs in ITP has also been supported by increasing evidence with encouraging efficacy. MSCs hold promise as a new approach to treat or even cure refractory ITP. Extracellular vesicles (EVs), as novel carriers in the “paracrine” mechanism of MSCs, are the focus of MSCs. Encouragingly, several studies suggested that EVs may perform similar functions as MSCs to treat ITP. This review summarized the role of MSCs in the pathophysiology and treatment of ITP.

## Introduction

Immune thrombocytopenia (ITP) is an acquired autoimmune disease characterized by isolated thrombocytopenia ((platelet count < 100,000/μL). Patients may be asymptomatic at presentation, or present with varying degrees of bleeding symptoms, such as ecchymosis, purpura, oral and nasal mucosa bleeding, genitourinary bleeding or increased menstrual bleeding. Rare and life-threatening bleeding, such as intracranial hemorrhage, occurs in very few patients [1, 2]. According to the duration, ITP can be divided into acute (< 3 months), persistent (3–12 months), and chronic (> 12 months). Although most children experience spontaneous remission, adult patients often show a chronic course. Irrespective of bleeding problems, patients with ITP often experience fatigue and impaired health-related quality of life [3].

The ultimate goal of treatment is to stop active bleeding and reduce the risk of future bleeding. The American Society of Hematological guidelines recommends observation, corticosteroids, and IVIG as first-line treatment. The second-line treatment includes thrombopoietin receptor agonist (TPO-RA), splenectomy, rituximab, immunosuppressants, etc.[4]. The pathophysiology of ITP is complex and remains incompletely clear. Clinically, the chronic ITP patient responds poorly to existing treatments or struggle to tolerate long-term drug therapy. Hence, there is an urgent need to further explore new treatments.

Mesenchymal stem cells (MSCs), also referred to as multipotent stromal cells or mesenchymal stromal cells, are multipotent, plastic-adherent cells that are present in multiple tissues, including bone marrow, adipose tissue, and Wharton jelly. Due to their low immunogenicity, pluripotent differentiation, and immunomodulatory ability, MSCs are widely used in a variety of autoimmune diseases. In recent years, increasing studies have found that impaired bone marrow mesenchymal stem cells (BMMSCs) play an important role in the pathogenesis of ITP. MSCs hold promise as a new approach to treat or even cure refractory ITP. Extracellular vesicles (EVs), as novel carriers in the “paracrine” mechanism of MSCs, are the hotspots in the research field. This review summarized the role of MSCs in the pathophysiology and treatment of ITP. The potential therapeutic effect of EVs derived from MSCs was also explored.

## MSCs in the pathogenesis of ITP

**Fig. 1:**
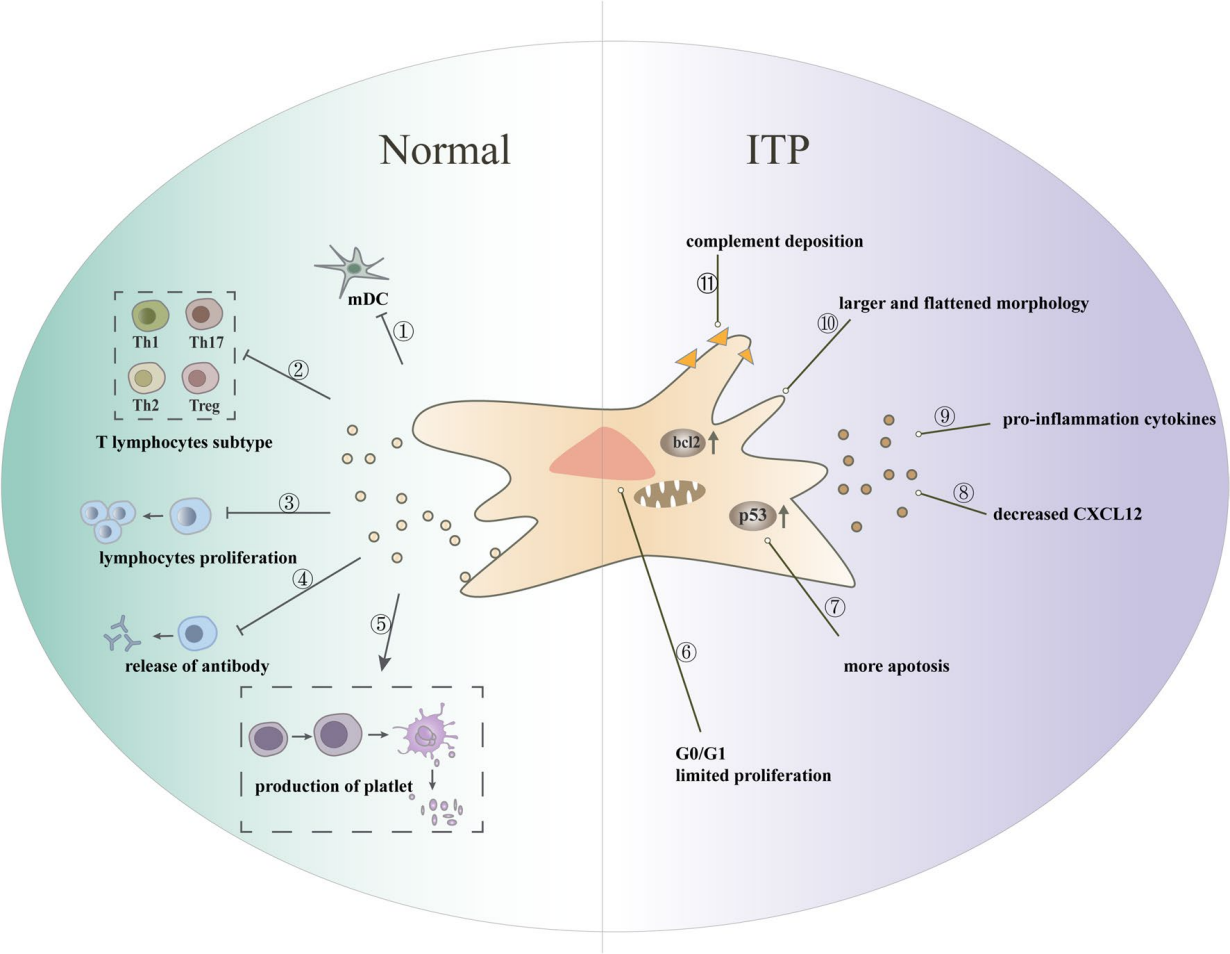
Phenotype and function of MSCs in healthy individuals and ITP patients. MSCs were involved in platelet production by interacting with immune cells, including ① inhibition of mDC maturation; ② regulation of the ratio of T lymphocyte subsets; ③ inhibition of lymphocyte proliferation; ④ inhibition of antibody release from B cells; and ⑤ promotion of platelet production from megakaryocyte maturation. The function of ITP-MSCs was impaired, mainly by: ⑥ increased G0/G1 cells with restricted proliferation; ⑦ increased apoptosis; ⑧ decreased concentration of CXCL12, which may contribute to reduced ability of megakaryocytes to migrate from the bone marrow sinus to the blood sinus; ⑨ increased release of pro-inflammatory cytokines; ⑩ broad and flattened appearance; ⑪ increased apoptosis of MSCs due to complement deposition. Ps: copyright permission is not applicable

The pathogenesis of ITP is complex, involving a variety of immune cells and immune factors, including immune-mediated platelet destruction [5], imbalance of T and B lymphocyte subsets [6], activation of the complement pathway [7], dysfunction of innate immune cells [8, 9], megakaryocyte dysfunction(impaired production, maturation, and apoptosis) [10–12], and abnormal bone marrow microenvironment [13]. However, most of the current treatments target a single pathogenic mechanism (e.g., IVIG mainly blocks Fc receptor-mediated phagocytosis, and splenectomy to avoid platelet destruction in the spleen), which may impede remission and cure in some patients. In recent years, many studies have identified MSCs in ITP (ITP-MSCs) with varying degrees of deficiency, which were involved in the development of ITP through multiple pathways (Fig. 1).

### Impaired proliferation and excessive apoptosis in ITP-MSCs

At the beginning of the twenty-first century, scholars in the ITP field turned their attention to bone marrow mesenchymal stem cells. Studies have shown that BMMSCs isolated from ITP patients or mouse models exhibited quantitative abnormality or functional deficiency [14–18]. The quantitative abnormality was thought to be related to their impaired proliferation and excessive apoptosis. First, ITP-MSCs expanded slowly and appeared flattened and larger, lacking the typical spindle shape [14–18], suggesting that the function of ITP-MSCs may be defective [18]. It is worth mentioning that ITP-MSCs were shown to have similar immunophenotype and capacity for trilineage mesenchymal differentiation as those from normal control [15, 18, 19], although prolonged differentiation time has been reported in a study [18]. Second, ITP-MSCs had a high rate of apoptosis in vitro culture, and both the extrinsic and the intrinsic apoptosis pathways were involved. The intrinsic pathway regulated the activity of the Bcl-2 family proteins that controlled the mitochondrial function; when proapoptotic factors, such as cytochrome C, were released from the mitochondria to the cytoplasm, the initiator caspase-9 activated, which ultimately caused a cascade response [14, 17]. In contrast, the extrinsic pathway was often dependent on transmembrane receptor-mediated interactions, such as Fas and FasL, or the direct activation of caspase-8 [14, 20]. In addition, an increase in G0/G1 cells, cell cycle arrest, and excess cellular senescence were also observed in ITP-MSCs [14].

### Potential mechanism of excess apoptosis in ITP-MSCs

Existing studies have well described the excess apoptosis and impaired proliferation in ITP-MSCs, while the specific signaling molecules and mechanisms were poorly understood. The expression of mRNAs and miRNAs in BMMSCs isolated from ITP patients and healthy controls was analyzed by microarray technology. Most of the differentially expressed genes were associated with signaling pathways related to cell proliferation, death, and survival [15]. The most notable of them was the impaired unfolded protein response (UPR) and reduced DNA transcription, which may cause a defective cellular stress response and ultimately lead to impaired survival and increased apoptosis of MSCs in an inflammatory environment [15]. Based on this finding, the team further screened for miR-98-5p, a microRNA significantly increased in ITP-MSCs, and further confirmed that it promoted apoptosis by inhibiting the IGF-2/PI3K/Akt pathway and upregulating p53 expression [21]. Previously, it has been demonstrated that increased p53/p21 expression in ITP-MSCs nuclei may be associated with MSCs apoptosis, senescence, and abnormal mitochondrial potentials [14]. These studies suggested that the p53 signaling pathway played an important role in ITP proliferation and apoptosis and that the exact mechanism of action needs to be further investigated.

Other Studies have suggested that complement deposition probably accounted for impaired ITP-MSCs [22–24]. The complement system is essentially a protein cascade reaction in the blood. Excessive complement deposition on platelets in ITP patients has been previously reported, and the antibody-induced complement activation pathway was an important mechanism of platelet clearance destruction [22–24]. A recent study found that complement c4d/c5b deposition on BMMSCs from ITP patients was associated with increased apoptosis, possibly due to complement-derived IL-1β/IL-1R/NF-κB, ERK1/2 and p38 MAPK signaling pathways [25], which has previously shown to be involved in MSC proliferation and migration differentiation [26].

### Abnormal immunomodulatory function of ITP-MSCs

In addition to the abnormal number of ITP-MSCs, its immunomodulatory function was also impaired. Cytokines, vital mediators of the immunomodulatory function of MSC, were altered in ITP. The pro-inflammatory cytokines (e.g., IFN-Ύ, TNF-ɑ, and IL-6) were increased in ITP-MSCs culture supernatants, whereas the anti-inflammatory cytokine, such as IL-4, was decreased [16, 19]. Secondly, ITP-MSCs had a lower ability to inhibit T lymphocyte proliferation in vitro [14, 16, 17], which may be related to the downregulation of suppressor genes involved in cell cycle control and the overexpression of caspase-9 [17]. In addition, the regulation of T lymphocyte subsets by ITP-MSCs was abnormal. Normal MSCs can suppress the ratio of Th1 and Th17 cells, induce differentiation of Treg cells, increase the ratio of suppressor T cells, and regulate the corresponding cytokine production. These effects of ITP-MSCs were compromised, which could be ameliorated by certain therapies, such as thalidomide, dexamethasone, and ATRA [18, 27].

Dendritic cells (DCs) are considered to be the main effector cells through which MSCs exert immunosuppressive effects. ITP-MSCs lost the ability to induce tolerance in mature dendritic cells (mDCs). mDCs, as the predominant antigen-presenting cells, can activate T cells and autoantibody-producing B cells, whereas tolerogenic mDCs block the proliferation of T cells activated by allogeneic mDCs. ITP-MSCs had a reduced ability to inhibit the expression of co-stimulatory molecules in mDCs and to promote the release of immunosuppressive cytokines [20]. In contrast, ITP-MSC-treated DCs produced more pro-inflammatory IL-12, suggesting a functional defect involved in peripheral immune intolerance to ITP. On the other hand, its ability to induce regulatory DC (regulatory DC inhibits proliferation and function of reactive T cells and induces Treg production) was lower, which may be related to the downregulation of the Notch-1/Jagge-1 signaling pathway [28]. In summary, it may conclude that ITP-MSCs shifted from a classical anti-inflammatory to a pro-inflammatory phenotype, which may eventually make ITP patients lose their immune tolerance.

### Impaired ability of ITP-MSCs to support platelet production

MSCs, as one of the important members of bone marrow, are the main regulators of bone marrow hematopoiesis and megakaryocyte function. Above all, MSCs can differentiate into mesenchymal cells such as vascular endothelial cells and osteoblasts to provide mechanical support for hematopoiesis [29]. Second, MSCs can express and secrete various cytokines to maintain hematopoietic progenitors and support megakaryocyte differentiation, such as chemokines ICAM-1,2 and VCAM-1, which can regulate the migration of megakaryocyte [19, 30]. In patients with ITP, the ability of MSCs to support megakaryocyte differentiation and platelet production was impaired, which may be related to the reduction of TNFAIP3 expression and overactivation of NF-kB signaling [22]. Furthermore, Nestin^+^ MSCs are the most important source of CXCL12 in the bone marrow; the nestin^+^ MSCs in ITP showed increased apoptosis, decreased populations, and reduced CXCL12 secretion, ultimately leading to impaired megakaryocyte distribution through CXCR4 and VEGFR1-mediated pathways [11].

## Therapeutic contribution of MSCs in ITP

It was believed that MSCs hold great therapeutic potential in rheumatoid arthritis, systemic lupus erythematosus, multiple sclerosis, ocular autoimmune diseases, etc. [31]. The possible mechanism was to alleviate excessive inflammatory responses by interacting with immune cells via cell–cell contact and secretion of soluble molecules. MSCs exerted immunomodulation by suppressing of abnormal proliferation and overactivation of T and B cells [31], upregulating tolerogenic dendritic cells [32], and regulating the differentiation and polarization of immune cell subpopulations [33]. As mentioned above, MSCs deficiency played an important role in the pathogenesis of ITP, which suggested that it may be a potential target for the treatment of ITP. Increasing studies confirmed this idea, and MSCs were expected to be a new strategy for the treatment of ITP. Following is a summary of the corresponding studies.

### In vitro cell culture

To the best of our knowledge, the study of MSCs in ITP began in 2008 when Zhiyong Qiu et al. found that MSCs can regulate the secretion of anti-platelet antibodies from splenocytes of ITP patients as well as inhibit the proliferation of platelet-reactivated T helper cells in vitro [34]. Since then, an increasing number of publications enriched the potential therapeutic role of MSC for ITP. In vitro, MSCs from bone marrow or adipose origin exhibited immunosuppressive effects and showed a concentration dependence. Clonal proliferation of lymphocytes can be found in ITP patients, but MSCs could inhibit the proliferation and co-stimulatory molecules CD40L and CD80 expression of these lymphocytes in vitro, especially for CD4^+^ T and CD19^+^ B cells [35]. In addition, MSCs can regulate the Th1/Th2 cell balance and promote Tregs, as shown by suppressing Th1-type cytokines, such as IFN-γ and TNF-α, and enhancing Th2-type cytokines, such as IL-4, ultimately restoring immune tolerance and alleviating ITP [36]. Other studies have shown that MSCs increased the proportion of CD8^+^CD28^−^ T suppressive cells and their suppression of T effective cells, which may correct the immune dysfunctions in ITP patients [27]. Most importantly, there was direct evidence that MSCs promoted megakaryocyte apoptosis and maturation, increased TPO production, and inhibited neo-platelet destruction and GPIIb-IIIa antibody production, ultimately increasing platelet counts [14, 37].

### Animal models

Several animal models of ITP were available, and their properties were well described in another review [38]. The passive immune model was relatively simple to operate and showed a rapid and time- and dose-dependent decrease in platelets after intraperitoneal injection of platelet antibodies into mice [39]. After transplantation of healthy human BMMSCs into ITP mice, platelet counts increased significantly and remained at high levels for a short period; meanwhile, bleeding symptoms, body weight, and splenic index were relieved [14, 40]. Similar effects were also proven in adipose-derived mesenchymal stem cells (AMSCs) [36].

Analysis of the immune status in mice by ELISA and flow cytometry revealed that: the level of anti-inflammatory cytokines in plasma, such as IL-10, IL-4, and TGFβ1, was increased after infusion; while pro-inflammatory cytokines, such as IFN-γ, IL-2, and IL-17, were downregulated, suggesting that MSCs can promote the shift of Th1 and Th17 cells to anti-inflammatory Th2 and suppressive Th3 cells in ITP mice. Consistent with in vitro experiments, ITP mice treated with MSCs showed a significant increase in the proportion of Treg cells in both peripheral blood and spleen, reduced production of anti-GPIIb-IIIa antibodies, and suppressed T lymphocyte proliferation [41].

Recently, the biodistribution of MSCs was of interest and was believed to be determined by cell source, cell size, immunological features, route of administration, and animal model [42]. Among these studies, a general biodistribution pattern of MSCs was available for the liver, spleen, and other inflamed tissues after the entrapment at lung capillary systems [42]. In addition, the distribution of MSCs to the bone marrow was reported by several studies [40, 43]. It is cheerful since the abnormal bone marrow microenvironment was an important cause of ITP. There were some strategies to improve the ability of homing to bone marrow of MSCs. It was reported that MSCs cultured on three-dimensional scaffolds culture had higher expression levels of extracellular matrix and bone marrow homing ability [40]. On the other hand, gene editing techniques were applied to enable cells to express specific molecules for homing to target tissues. Sackstein et al. engineered the active E-selectin (expressed by specialized marrow vessels and related to cellular recruitment) ligand on the surface of plastic-adherent MSCs, resulting in efficient homing to bone marrow [44]. However, the fate of MSCs still needs to be further explored in animal models of ITP or patients, since the biodistribution is influenced by the immune status of the recipient.

### Clinical efficacy

Chronic refractory ITP has been a dilemma for clinical treatment. Encouraged by the clinical efficacy of MSCs in autoimmune diseases, clinicians have begun to experiment with MSCs for the treatment of ITP. Encouraged by the successful application of MSCs in other autoimmune diseases, clinicians began to use MSCs for ITP [37, 45, 46], as summarized in Table 1. The utilization of MSCs in patients with ITP was first reported in 2009 in a patient diagnosed with chronic refractory ITP who continued to relapse despite multiple treatments (including glucocorticoids, immunosuppressants, splenectomy, and autologous peripheral blood stem cell transplantation). After receiving AMSCs infusion, the patient's platelets gradually increased to normal after 2 months, and sustained remission for 55 months with no infusion-related adverse effects observed [47]. Subsequently, the medical center applied AMSCs to successfully treat five cases of refractory ITP, with all cases achieving sustained remission (remission duration 8.5–19 months). Notably, three of these cases relapsed within 6 months of remission, and a second remission was achieved after reinfusion of AMSCs [45]. In addition to AMSCs, human umbilical cord mesenchymal stem cells (hUCMSC) have also been reported for the treatment of refractory ITP. In summary, MSCs have the following characteristics for the treatment of ITP: effective in refractory ITP that is resistant to existing treatments; increasing platelet counts within a short period; achieving clinical remission in most cases after a single administration; long duration of remission with the possibility of clinical cure. However, the current encouraging results come from bold attempts in small cohorts, and it is difficult to avoid publication bias. Further, the heterogeneity of participants, interventions, and outcomes makes it challenging to combine and compare results across studies. It is expected that more reports, preferably with large-scale multicenter data, will help further evaluate the safety and efficacy of MSCs for ITP.

**Table 1:** The clinical efficacy of MSCs for treatment of ITP patients

No	Age/gender (years)	Duration (months)	Previous treatment	MSC type	Dose (10^6^/kg)	Times	Platelet counts 10^9^/L		Time to response (days)	Overall response	Response duration (months)	Refs
							Before	After				
1	21/M	43	P,V,A,C,Ig,S,Cy,PBSCT	AMSC	2	1	9	51	11	Yes	55	[43]
2	34/F	71	P,V,A,C,Ig,S,Cy	AMSC	2	1	12	92	11	Yes	19	
3	45/M	62	P,Ig,A,D,De,S	AMSC	2	1	5	103	12	Yes	13	
4	32/F	53	P,D,V,A,Ig,S	AMSC	2	2	11	91	11	Yes	8.5	
5	29/M	37	P,D,V,A,S,Cy	AMSC	2	1	15	104	17	Yes	12	
6	22/F	74	P,De,V,A,S	AMSC	2	2	3	45	12	Yes	10	
7	38/M	14	P,A,S,V	AMSC	2	2	10	99	16	Yes	8.7	
8	26/M	43	P,V,Cy,Ig,S	UCMSC	1	1	8	56	7	Yes	24	[44]
9	49/F	71	P,V,Cy,Ig	UCMSC	1	2	9	94	13	Yes	18	
10	54/F	62	P,Ig,Cy,De,S	UCMSC	1	2	5	103	16	Yes	13	
11	50/F	120	P,Ig,V	UCMSC	1	2	3	56	14	Yes	13	
12	64/M	54	P,D,Ig,S,De,C,	UCMSC	0.4	1	1	33	7	Yes	3	[36]
13	66/F	49	P,D,Ig,S,R,	UCMSC	0.4	1	12	59	14	Yes	9	

F, female; M, male; P, prednisone; V, vincristine; A, azathioprine; C, cyclophosphamide; Ig, intravenous immunoglobulins; S, splenectomy; Cy, cyclosporin A; PBSCT, peripheral blood stem cells transplantation; D, danazol; De, dexamethasone; R, rituximab; AMSC, adipose tissue-derived mesenchymal stem cell; UCMSC, umbilical cord mesenchymal stem cells; response: platelet count ≥ 30,000/µL and at least doubling baseline

## MSC-EVs: the future focus and strategy for the treatment of ITP

Although encouraging results have been achieved with small-scale clinical applications of MSCs, the publications on the clinical use of MSCs in ITP were not as prolific as expected, possibly due to clinicians’ concerns and caution about the potential side effects of “cell therapy.” The relatively large size of MSCs would result in mechanical entrapment at lung capillary systems after intravenous administration, a phenomenon referred to as the pulmonary first-pass effect, which potentially decreased the number of cells available for target tissues [48]. Another inescapable concern was that large MSCs may be associated with vascular occlusions and embolisms [49]. In addition, the homing ability of MSCs to damaged organs was low after intravenous administration and the mechanisms were less well understood. On the other hand, numerous evidence suggested that MSCs act primarily through paracrine. Therefore, current research is dedicated to the use of active substances secreted by MSCs to replace their biological functions and avoid side effects of cell therapy, with EVs standing out.

EVs are lipid-bound vesicles secreted by cells into the extracellular space, which are known to facilitate intercellular communication in diverse cellular processes such as immune responses and inflammation by transferring cargoes [50]. Mesenchymal stem cell-derived extracellular vesicles (MSC-EVs) are the highlights in stem cell research and are promising as a new strategy for the cell-free therapy of autoimmune diseases. As mentioned above, the pathogenesis of ITP involved multiple mechanisms. Current studies suggested that MSC-EVs, just like MSCs, can interact with immune cells and modulate the immune response. First, MSC-EVs could inhibit autoreactive lymphocyte proliferation and induce the secretion of anti-inflammatory cytokines, partly by expressing tolerogenic molecules, such as PD-L1 and galectin-1 [51]. Next, MSCs exhibited strong inhibition of B cell proliferation and immunoglobulin secretion, in which EVs play a major role [52]. In addition, the effect of MSC-EVs in the polarization of M2 macrophages was identified, of which the anti-inflammatory properties are potentially effective in inflammation diseases [53]. On this basis, it is reasonable to believe that MSC-EVs can similarly treat ITP. In 2021, two publications studied the role of MSC-EVs in ITP in vitro and in vivo, respectively [54, 55]. BMMSCs-derived exosomes significantly reduced the Th17/Treg ratio of ITP patients in vitro; the possible mechanism was that miR-146a-5p, highly expressed in the exosomes of BMMSCs and delivered to CD4^+^ T cells, inhibited IL-1R-associated kinase-1 (IRAK) expression to regulate Th17/Treg imbalance, ultimately leading to lower IL-17 levels and higher IL-10 and TGFβ levels, suggesting that exosomal miR-146a-5p may be a potential target for the treatment of ITP [54]. The role of microRNA in MSC-EVs was also confirmed in another study. AMSCs modified with miR-199a-5p can secrete miR-199a5p into EVs. MiR-199a-5p highly expressed EVs inhibited Th17 cell differentiation and elevated platelet counts both in vitro and in vivo in mice [55]. In summary, MSC-EVs deliver active substances, including miRNAs, that exert immunomodulatory effects and hold promise as a new strategy for the treatment of ITP. Unfortunately, more effort is still needed to explore the potential role of MSC-EVs or their specific cargoes, such as proteins, on ITP.

## Conclusion and prospects

ITP is a common autoimmune disease with a good prognosis in most patients, but some patients remain refractory. Research in recent decades has been devoted to exploring pathological mechanisms and novel therapeutic agents. Among them, the emergence of TPO-RAs is a milestone in this field, changing the traditional regimen of ITP treatment relying on immunosuppression [56, 57]. However, most patients need to take TPO-RAs continuously to maintain platelet counts, incurring long-term financial costs and side effects. Research on mesenchymal stem cells in ITP has enriched the pathological mechanisms and provided hope for the cure of ITP. The therapeutic potential of MSCs for ITP has been validated in vitro and animal models. Furthermore, encouraging efficacy has also been achieved with MSCs in the clinical treatment of refractory ITP, although the sample size was small. Unfortunately, MSCs have not been as intensively studied in ITP as in other autoimmune diseases. This may be because that ITP is mostly a benign disease and the clinical application is limited by the shortcomings of the cell therapy. It is promising that MSC-EVs can preserve immunomodulation while avoiding the side effects of cell therapy. The role of MSC-EVs in ITP has been elucidated in several recent papers, but there is still much space for improvement.

MSC-EVs are the next direction for stem cell research but are still far from being applied to the clinic with some concerns and questions requiring answers. First, the complexity and the heterogeneity of patients are far from being simulated by animal models. There were many controversies regarding the timing, dose, and frequency of administration of MSCs and EVs. Secondly, although the International Society for Cellular Therapy [58] and the International Society for Extracellular Vesicles [59] have standardized the minimal criteria of MSCs and EVs, respectively, their biological properties are still largely influenced by many factors. Heterogeneity has restricted the reproducibility of studies and the comparability of results across studies. Recent remarkable advances in the single-vesicle analysis have made it possible to overcome these limitations and facilitated the "precision" era of EV research [60]. Finally, novel strategies are required to enhance the capability of survival, homing to the site of damage since the dose or frequency of MSCs and EVs cannot be increased infinitely. In conclusion, these innovative technologies and affiliated studies pave the way toward unraveling the complex pathogenesis and novel therapy of ITP. MSCs have been successfully applied in treating ITP, of which EVs were considered important mediators, and further research may encourage their wider use, ultimately making a cure for ITP possible.

## Data Availability

Data sharing does not apply to this article as no datasets were generated or analyzed during the current study.

## Abbreviations

ITP: Immune thrombocytopenia
MSCs: Mesenchymal stem cells
EVs: Extracellular vesicles
BMMSCs: Bone marrow mesenchymal stem cells
ITP-MSCs: MSCs in ITP
AMSCs: Adipose-derived mesenchymal stem cells
